# Ion exchange-assisted surface passivation toward highly stable red-emitting fluoride phosphors for light-emitting diodes

**DOI:** 10.1038/s41598-024-65169-z

**Published:** 2024-06-26

**Authors:** Xumian Qiao, Hassan Siddique, Xinhua Li, Song Zhu, Sifei Liu, Maomao Gu, Haoyu Yang, Donghui Zhang, Qiang Zhao

**Affiliations:** 1 School of Mathematics and Physics, Anhui Jianzhu University, Hefei, 230601 China; 2 Anhui Research Center of Generic Technology in New Display Industry, Hefei, China; 3 National University of Modern Languages, Islamabad, Pakistan; 4 Govisionox Technology Company, Bengbu, China

**Keywords:** Materials science, Materials for optics, Lasers, LEDs and light sources

## Abstract

A facile and environmentally friendly ion exchange-assisted surface passivation (IASP) strategy is presented for synthesizing red emitting Mn^4+^-activated fluoride phosphors. A substantial, pristine Mn^4+^-free shell layer, applied as a coating to Mn^4+^ doped potassium fluorosilicate K_2_SiF_6_:Mn^4+^ (KSFM) phosphors, enhances both water resistance and luminescence efficiency. The stability test of fluoride in water at ambient temperature and boiling water demonstrates that IASP-treated KSFM phosphors are highly water resistant. Furthermore, both the negative thermal temperature (NTQ) fitting results and the photoluminescence (PL) decay confirm that the IASP process effectively passivates surface defects, leading to enhanced luminescence performance. The maximum internal quantum yield (QY_i_) of the IASP-KSFM phosphor is 94.24%. A white LED realized a high color rendering index (CRI) of 93.09 and luminous efficiency (LE) of 149.48 lm/W. This work presented a novel technique for the development of stable fluoride phosphors and has the potential to increase the use of KSFM phosphors in plant supplementary lighting systems and white light-emitting diodes.

## Introduction

Phosphor-converting white light-emitting diodes (pc-WLEDs) are emerging as the forefront technology for cost-effective and space-efficient lighting solutions, driven by their outstanding energy efficiency, extended lifespan, compact design, and environmentally friendly qualities^1–4^. Blue LED chips and YAG:Ce^3+^ yellow-emitting phosphor are commonly used pc-WLEDs. Since the white emission from blue LED chips and YAG:Ce^3+^ yellow-emitting phosphor lacks red spectral emission, this scheme cannot achieve the ideal lighting effect, resulting in high CCT and poor Ra^5–8^.

A feasible way to tackle the high CCT and poor Ra is to add a red-emissive phosphor. Mn^4+^ doped fluoride phosphors, such as A_2_MF_6_:Mn^4+^ (A = K, Na, Li, Cs, Rb, M = Si, Ge, Ti, etc.)^9–11^, A_3_BF_6_:Mn^4+^ (B = Al, Ga, etc.)^12,13^, can absorb a wide range of blue light and emit a narrow band of red light with high color purity and high quantum efficiency. Furthermore, this type of fluoride phosphors exhibits the advantages of simple synthesis and low costs, which makes them promising candidates for red phosphorescence in WLEDs^14,15^. However, the [MnF_6_]^2−^ group on the fluoride phosphors surface is easily hydrolyzed into non-luminous oxides and hydroxides in a moisture environment^16–18^. This drawback causes deterioration of LEDs during long-term operation. Therefore, it is necessary to substantially improve the moisture resistant properties of the fluoride phosphors.

In recent years, many solutions have been proposed to improve the moisture resistance problem of the fluoride phosphors. One common reported strategy is to construct the heterogeneous organic or inorganic moisture-resistant shells to protect active Mn^4+^ from the external environment^19–21^. For example, Zhou et al. coated the octadecyltrimethoxysilane (ODTMS)^22^ to form a super hydrophobic surface with good water resistance and thermal stability. Meanwhile, some inorganic material such as CaF_2_^23^, Al_2_O_3_^24,25^, SiO_2_^26^ and GQDs^27^ were proposed to construct water-resistant shells. These protective layer shells can effectively improve the moisture resistance of the fluoride phosphors. Nonetheless, there are large differences in physicochemical properties between the protective shell and the core fluoride phosphors. When subjected to repeated temperature fluctuations, local interface defects are prone to be introduced, resulting in the enhanced nonradiative decay probability and the reduced luminescence efficiency of the fluoride phosphors.

Another strategy is to construct a homogeneous fluoride shell on the phosphor surface. A water-resistant Mn^4+^-free or Mn^4+^-rare A_2_XF_6_ fluoride matrix shell is proposed to isolate the [MnF_6_]^2−^ clusters from water. The moisture resistance of the A_2_MF_6_:Mn^4+^ phosphors core is enhanced^28,29^. Oxalic acid (H_2_C_2_O_4_)^30^, hydrogen peroxide (H_2_O_2_)^31^, and other weak reducing agents have been used to remove Mn^4+^ on the phosphor surface. This method can significantly improve the water resistance without reducing the luminescence intensity of the fluoride phosphors. But the reduced protective shells are too thin, and thereby the Mn^4+^ ions below the passivation layer are prone to be hydrolyzed in an extreme working environment such as high temperature and humidity. To further increase the thickness of the Mn^4+^-free shell layer, an ion exchange-promoted surface reduction technique was recently proposed^32^. In this method, fluoride phosphors are placed in its saturated solution for ion exchange. Meanwhile, a reducing agent sodium nitrite (NaNO_2_) was added to reduce the Mn^4+^ on the phosphor surface. While this approach successfully forms a thick and Mn^4+^-free shell and enhances the stability of the phosphor matrix, it is environmentally unfriendly because it uses nitrite materials as the reducing agent. Hazardous gas (nitrogen dioxide) emits during the reaction process, which limits its potential for widespread commercial use. In order to produce phosphors with excellent performance, a simple, efficient, and environmentally friendly surface modification method still needs to be developed.

In this paper, we proposed a simple and environmentally friendly ion exchange-assisted surface passivation (IASP) strategy to enhance the moisture resistance and luminescence efficiency of Mn^4+^ doped potassium fluorosilicate K_2_SiF_6_:Mn^4+^ (KSFM) phosphors. Firstly, fluoride phosphors are placed in a saturated solution of K_2_SiF_6_ (KSF) to undergo ion exchange. When the solution reaches a dynamic equilibrium state of dissolution and crystallization, an environment-friendly potassium hypophosphite (H_2_KO_2_P) is used as a reducing agent to construct a thick shell layer without Mn^4+^ on the surface of core phosphors. This method effectively improves the water resistance and luminescence efficiency of the phosphors without releasing any toxic gases during the entire passivation process. This safe and efficient modification strategy provides a new approach for developing stable and efficient fluoride phosphors, and it can also promote the application of KSFM phosphors in the field of WLEDs.

## Experiment section

The experiment details are in the Supporting Information. For comparison, three types of phosphors were synthesized. KSFM represents the bare phosphors without any surface treatments. SP-KSFM represents the phosphors treated only by the surface passivation agent (H_2_KO_2_P solution). IASP-KSFM represents the phosphors treated by IASP strategy.

## Results and discussion

### Structure, morphology and composition analysis

**Figure 1:**
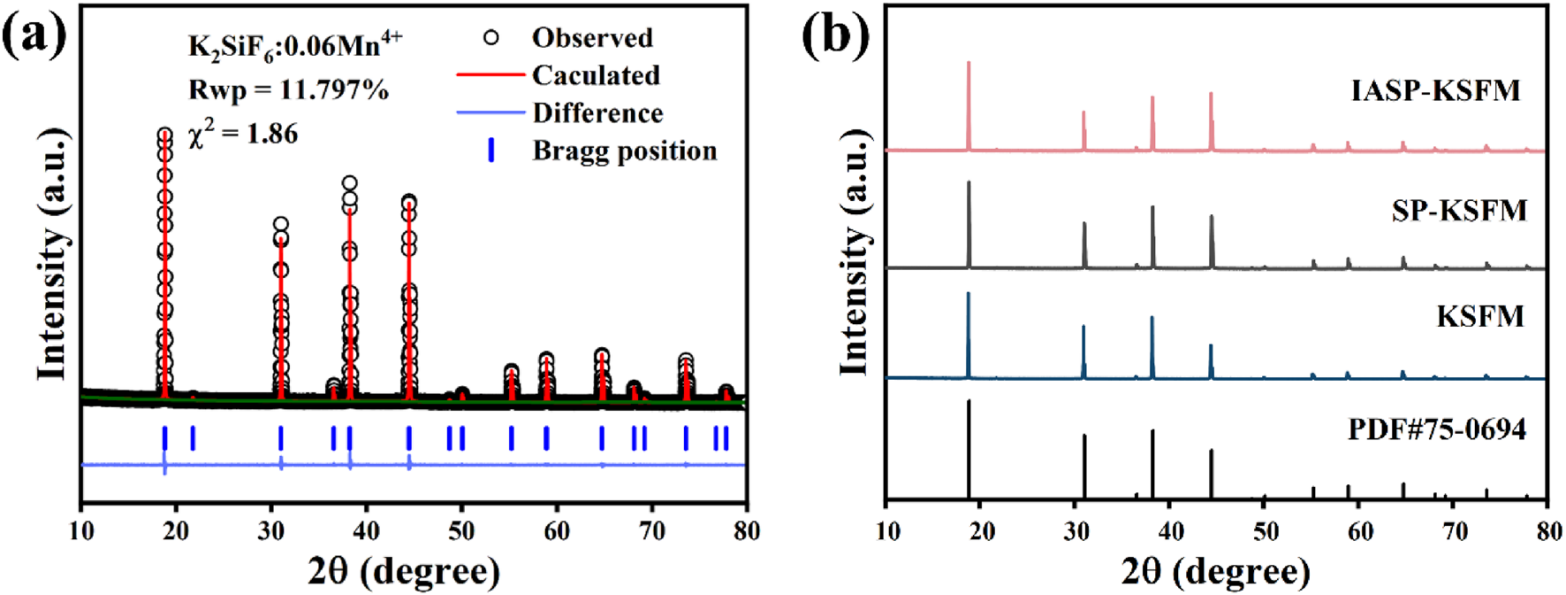
(**a**) X-ray Rietveld refinement of the KSFM (0.06 Mn^4+^) phosphors. (**b**) X-ray diffraction patterns of the KSFM (0.06 Mn^4+^), the SP-KSFM (0.06 Mn^4+^) and IASP-KSFM (0.06 Mn^4+^) phosphors, respectively.

The Rietveld refinement analyses of powder XRD profiles of the representative KSFM (0.06 Mn^4+^) conducted by the general structure analysis system II (GSAS II) method are demonstrated in Fig. 1a. All diffraction peaks in the diffraction pattern are indexed to the standard patterns of cubic K_2_SiF_6_ (PDF Card no. 75-0694), with a good fitting result of R_wp_ = 11.7%, χ^2^ = 1.86. This indicates the successful synthesis of pure-phase phosphors as intended.

Figure 1b shows the XRD patterns of the as-synthesized K_2_SiF_6_ (0.06 Mn^4+^) (KSFM), KSFM phosphors treated by the surface passivation strategy (SP-KSFM) and KSFM phosphors treated with ion exchange-assisted surface passivation strategy (IASP-KSFM). No impurity peaks are detected, indicating that all soluble contaminants are removed and the pure phase KSFM phosphors are successfully synthesized by the proposed methods. The lattice parameters of the samples are shown in Table S1. The lattice parameter of KSFM phosphor is 8.1775 Å, which is slightly larger than that of IASP-KSFM (8.1432 Å). This can be attributed to the smaller radius of Si^4+^ ions (CN = 6, r = 0.40 Å) than that of Mn^4+^ ions (CN = 6, r = 0.53 Å). After the KSFM phosphors are treated by the IASP strategy, the Mn^4+^ ions are exchanged by Si^4+^ ions, leading to the contraction of the cell volume of IASP-KSFM^32^.

**Figure 2:**
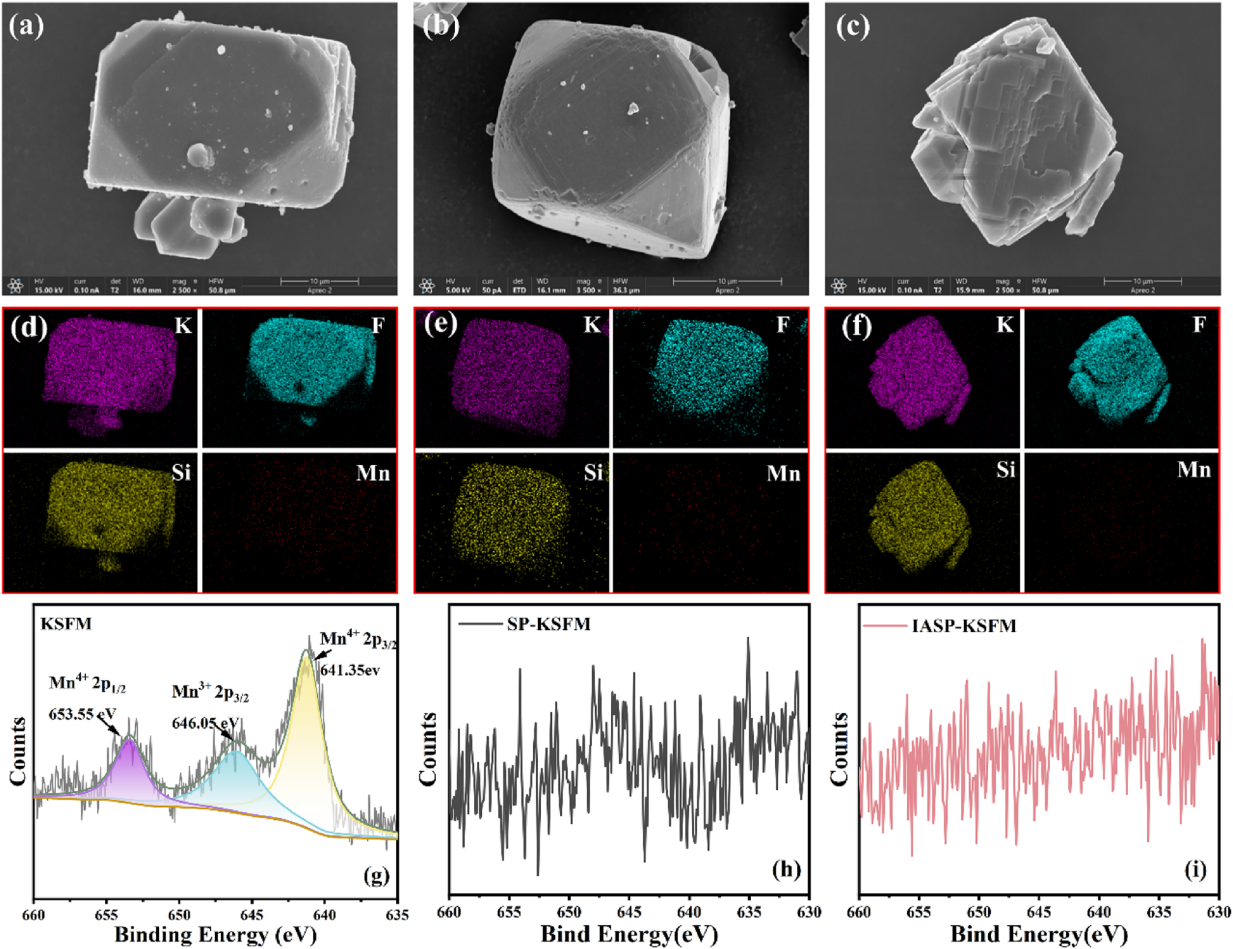
SEM images of three samples, (**a**) KSFM, (**b**) SP-KSFM, (**c**) IASP-KSFM. (**d**–**f**) is the EDS mappings corresponding to the three samples. (**g**–**i**) High-resolution XPS spectra of Mn 2p in three samples.

Figure 2a–c shows the SEM micrograph of KSFM (a), SP-KSFM (b) and IASP-KSFM (c). It can be seen the KSFM phosphor shows a smooth and regular morphology. After SP and IASP treatment, the edges and corners of the phosphor become slightly dissolved, and the phosphor surface becomes rough. This phenomenon could be attributed by the exchange between the IASP-KSFM phosphor and the potassium flurosilicate solution, as well as the corrosion of the potassium hypophosphite solution. The EDS maps of the KSFM samples are displayed in Fig. 2d–f both before and after the SP and IASP approach treatment. As expected, the elements K, Si, and F are uniformly distributed on the surfaces of the three phosphors, whereas the surface content of the Mn element in the IASP-KSFM sample is nearly undetectable, significantly lower than that observed in the KSFM sample.

The complete X-ray photoelectron spectroscopy (XPS) of KSFM, SP-KSFM and IASP-KSFM phosphors are shown in Fig. S1. Clear signal peaks of K, Si, F elements can be observed. We also analyzed the high-resolution Mn 2p XPS spectra of the KSFM, SP-KSFM, and IASP-KSFM phosphors in order to confirm the impact of surface treatment on the Mn concentration at the KSFM surface. As shown in Fig. 2g, in the KSFM sample, three obvious signals of Mn^4+^ 2p_3/2_, Mn^3+^2p_3/2_ and Mn^4+^ 2p_1/2_ were detected in the range of 655–650 eV, 650–645 eV and 645–640 eV^33,34^. Meanwhile, weak peaks corresponding to Mn 2p_3/2_ and Mn 2p_1/2_ signals were also detected in the SP-KSFM sample as shown in Fig. 2h. This implies that Mn is still present at the surface and Mn cannot be entirely removed using the SP technique. In contrast, no significant Mn 2p signals were identified in the IASP-KSFM phosphor as shown in Fig. 2i. This illustrates the successful application of the IASP technique in eliminating Mn^4+^ activator ions from the surface of the fluoride phosphor, thereby establishing a Mn^4+^-free protective layer. The obtained result aligns consistently with the EDS result analysis.

The XPS and SEM data demonstrated that a Mn^4+^-free shell had formed on the surface, effectively shielding the internal Mn^4+^-activated ions. The formation mechanism of the Mn^4+^-free shell can be explained by the following chemical reaction equations^35^:

1 $${K_{2} Si_{(1 - x)} Mn_{x} F_{6} (s) \to (1 - x)[SiF_{6} ]^{2 - } (aq) + 2K^{ + } (aq) + x[MnF_{6} ]^{2 - } (aq)}$$

2 $${K_{2} Si_{(1 - x)} Mn_{x} F_{6} (s) + [SiF_{6} ]^{2 - } (aq) + 2K^{ + } (aq) \leftrightharpoons K_{2} Si_{(1 - x)} Mn_{x} F_{6} (s)@K_{2} SiF_{6} (s)}$$

3 $${[MnF_{6} ]^{2 - } (aq) + H_{2} PO_{2}^{ - } (aq) + 4H^{ + } + H_{2} O \to Mn^{2 + } (aq) + 6HF(aq) + H_{2} PO_{3}^{ - } (aq)}$$

**Figure 3:**
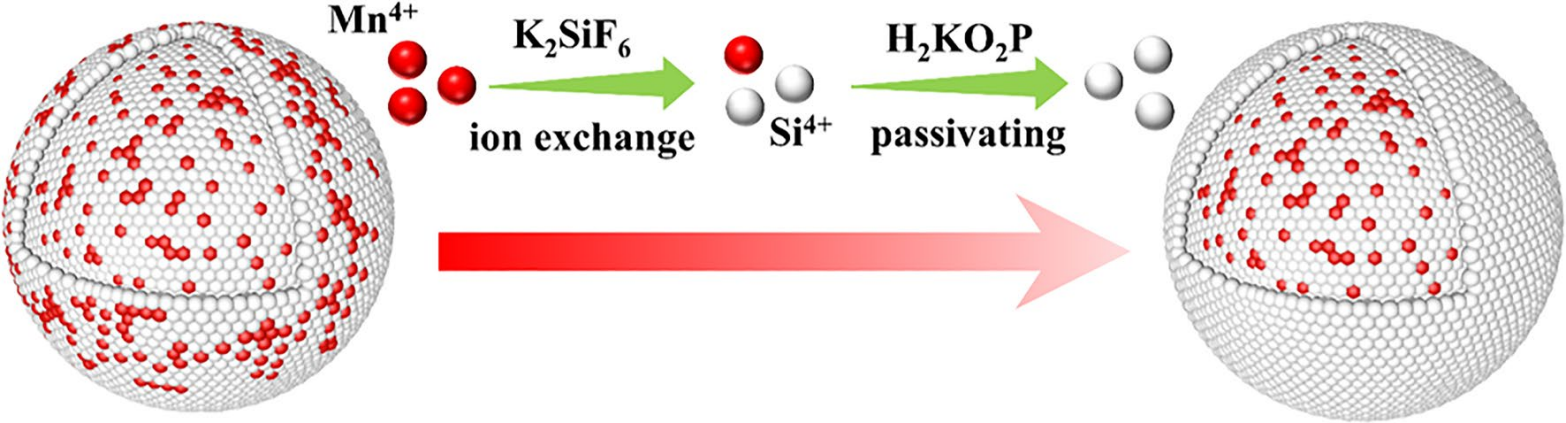
An illustration of the construction of Mn^4+^-free shell through IASP strategy.

Firstly, KSF powders are added in a high concentration HF solution to form a saturated KSF solution. Then, K_2_Si_(1−*x*)_Mn_*x*_F_6_ phosphors are dissolved in the saturated KSF solution, the K^+^, [SiF_6_]^2−^ and [MnF_6_]^2−^ luminescent groups are ionized, as shown in the chemical reaction Eq. (1). The [MnF_6_]^2−^ group in the surface layer of the phosphor is constantly exchanged into the solution. Since the KSF solution is in a saturated state, the fluoride crystals are in a stage of dynamic equilibrium. The [SiF_6_]^2−^ group will combine with K^+^ to construct KSF protective layer and form the KSFM@KSF core–shell structure represented in Eq. (2). When the [MnF_6_]^2−^ group in the solution reaches a certain concentration, the [MnF_6_]^2−^ group will re-enter the KSF crystal surface, which increases the difficulty in achieving a true Mn^4+^-free shell. In order to construct the shell without Mn^4+^-activated ions, ${\text{H}}_{2} {\text{PO}}_{2}^{ - }$ ions are introduced. As shown in Eq. (3), the [MnF_6_]^2−^ groups are reduced to Mn^2+^ ions by ${\text{H}}_{2} {\text{PO}}_{2}^{ - }$, which removes the Mn^4+^ in solution and prevents the re-doping of [MnF_6_]^2−^ in the KSF shell. The whole process is described in Fig. 3.

To verify the effectiveness of the ion exchange reactions shown in Eqs. (1)–(3), we further analyzed the radial element distribution in the KSFM phosphors treated by SP and IASP process. Then Energy dispersive spectroscopy (EDS) analysis was performed to analyze the Mn element content at different depths from the surface shown in Fig. S2. We found that the SP-KSFM sample still contained a certain amount of Mn at the surface. To further verify the thickness of the Mn^4+^-free shell, the focused ion beam (FIB) technique was used to obtain the cross sections of the IASP-treated KSFM particles. EDS mapping images of K, Si, F, and Mn were performed on the cross section by EDS. As shown in Fig. S2, we didn’t observe the Mn elements at the surface of the IASP treated sample. After treatment for 20 min, Mn elements were not detected at the depth of 0.81 μm from the surface, indicating that the IASP technology can effectively construct a Mn^4+^-free shell layer. At the same time, according to the results reported by Ruan et al.^32^, it took 40 min to obtain a 0.6 μm Mn^4+^-free shell layer by the NaNO_2_ passivation solutions. Compared to their technology, our IASP strategy is more efficient in constructing a thick Mn^4+^-free shell layer at micron level. Notably, in our approach of IASP, we selected H_2_KO_2_P as the passivation agent, which is commonly used in pharmaceuticals and food processing fields. In the entire IASP process, no toxic gas or harmful substances were produced. The reaction remnants can be recycled and treated by simple chemical precipitation.

4 $${3Ca^{2 + } 2OH^{ - } + H_{2} PO_{3}^{ - } + H^{ + } \to 2CaHPO_{3} \downarrow + Ca[H_{2} PO_{3} ]_{2} \downarrow + 6H_{2} O}$$

Therefore, the proposed IASP strategy is a safe, environmentally friendly, and easy-to-operate surface modification method for Mn^4+^ doped fluoride fluorescent powders.

### Moisture resistance performances

**Figure 4:**
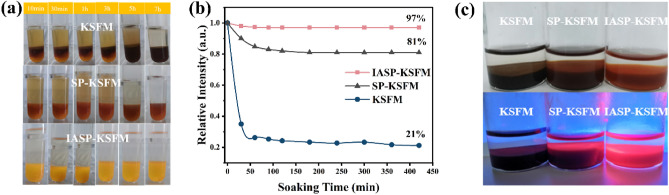
(**a**) Photographs of phosphors soaked in deionized water treated with different strategies. (**b**) The trend of luminescence intensity in the water resistance test for each sample. (**c**) Photographs of each sample before and after boiling under natural (top) and ultraviolet light (bottom).

The time-dependent color shift of the samples was used to examine the impact of IASP on the moisture-resistant properties of KSFM phosphors. The KSFM, SP-KSFM and IASP-KSFM samples were immersed in deionized water (3 ml) for time dependent color change of the samples shown in Fig. 4a,b. The KSFM and SP-KSFM samples quickly changed from yellow to brown after 10 min, while the IASP-KSFM samples remained yellow in water for 420 min. The PL intensity of the IASP-KSFM sample remained at 97% after 420 min of soaking, which was higher than that of KSFM (21%) and SP-KSFM (81%). In the moisture environment, the surface [MnF_6_]^2−^ group would be easily hydrolyzed into mixed-valence Mn oxides and hydroxides. This darkens the phosphor body color and weakens the PL emission intensity of bare KSFM sample. For SP-KSFM sample, Mn^4+^ on the phosphor surface is removed by the reducing agent, which significantly improves the water resistance of the phosphor. But as demonstrated in Fig. S2, the Mn^4+^-free shell is too thin to maintain a long-term protective effect. As comparably, the IASP process can provide a thick and Mn^4+^-free KSF shell. This results in the good and stable water resistance effect of IASP-KSFM sample.

The boiling tests were further performed to evaluate the moisture-resistance property under extreme working conditions. A video of the boiling test is placed in the supporting information. After being boiled for 20 min, the bare KSFM and SP-KSFM phosphors immediately changed from yellow to dark brown, while the IASP-KSFM sample still remained bright yellow and emitted strong red light under blue light irradiation shown in Fig. 4c. Accordingly, the PL intensities of the KSFM, SP-KSFM and IASP-KSFM samples retained approximately 10%, 35% and 82% of their initial values, respectively (Fig. S3). This indicates that IASP treatment can significantly improve the moisture resistant capabilities of KSFM phosphors. As shown in Fig. S2, the IASP strategy can exchange the Mn^4+^ ions in the deeper part of the KSFM phosphor to form a KSF shell. Meanwhile, the reducing agent H_2_KO_2_P is added to avoid the redoping of Mn^4+^ ions in the KSF shell. Therefore, a thick Mn^4+^-free shell layer is formed on the surface of the IASP-KSFM phosphor. Herein, the IASP-KSFM phosphor have the better moisture resistance than that of SP-KSFM samples, in which only a thin protective shell is formed. Due to the same physicochemical properties between inner phosphors and protective layers, the IASP process would not sacrifice the luminescence efficiency while maintaining a long-term protective effect in an extreme hydrolytic environment such as high temperature and humidity.

### Luminescent properties

**Figure 5:**
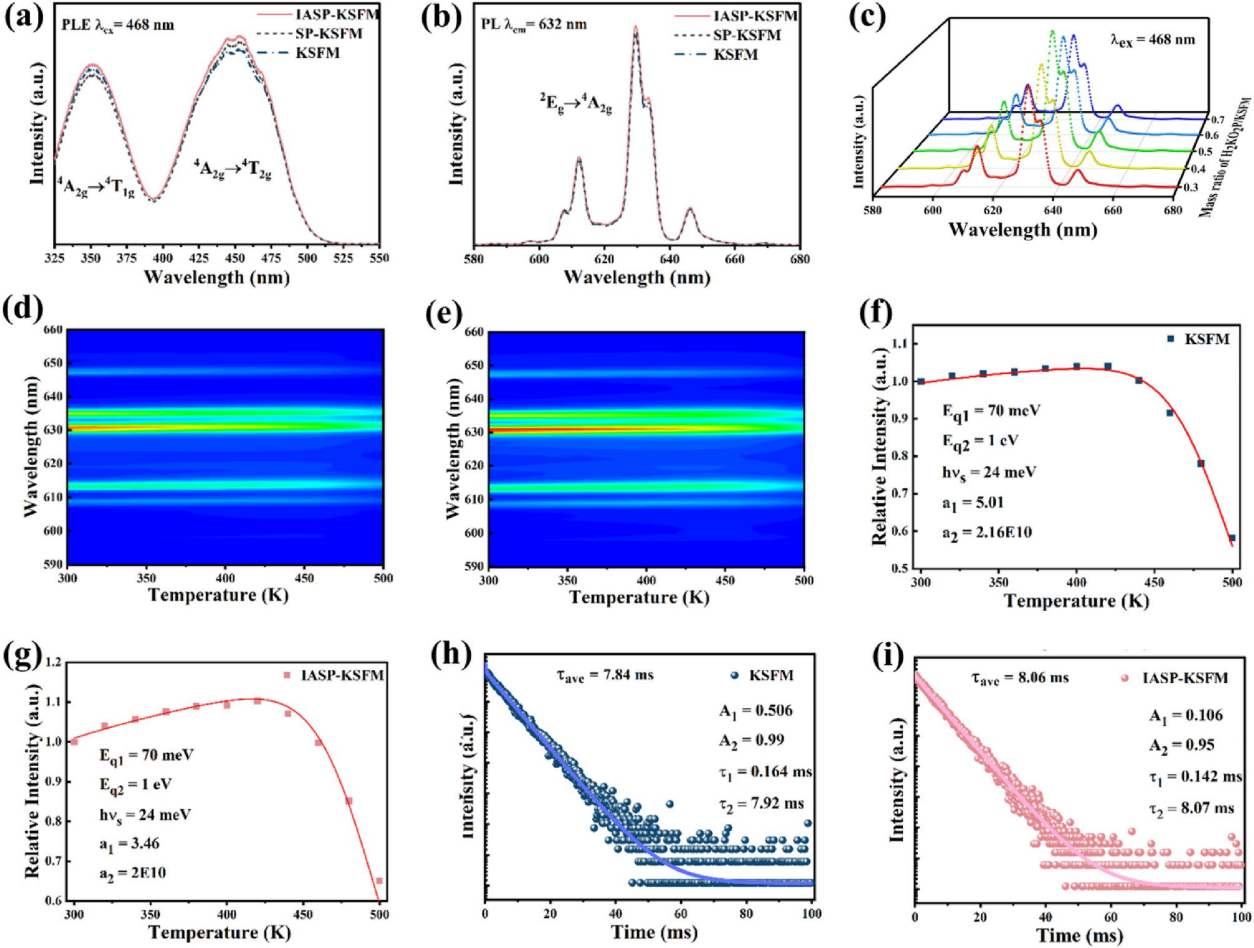
Luminescence properties of KSFM, SP-KSFM and IASP-KSFM. (**a**) PLE spectra, (**b**) PL spectra, (**c**) PL spectra of IASP-KSFM samples treated by variant mass ratio of H_2_KO_2_P/KSFM. (**d**,**e**) Temperature-dependent emission spectra in terms of contour map, (**f**,**g**) temperature-dependent relative integrated PL intensity in the range of 580–680 nm. The red solid lines represent the best-fit results calculated using Eq. (4) with different $a_{1}$, $a_{2}$, and $I_{0}$ values (α = 0, $E_{q1}$ = 70 meV (j = 1), $E_{q2}$ = 1 eV (j = 2), and $hv_{s}$ = 24 meV). (**h**,**i**) Luminescent decay lifetime curves of the samples. (**h**) KSFM (0.06 Mn^4+^), (**i**) IASP-KSFM (0.06 Mn^4+^).

Figure 5a,b show the PLE and PL spectra of KSFM and IASP-KSFM at room temperature. It can be observed that the shapes and emission peak positions of the PLE/PL spectra of the three samples are basically consistent. Two broadband excitation peaks at 360 nm and 468 nm can be observed in the ultraviolet and blue light regions of 300–500 nm, which belong to the spin-allowed transitions of ^4^A_2g_ → ^4^T_1g_ and ^4^A_2g_ → ^4^T_2g_^36^. The electrons on ^4^T_1g_ or ^4^T_2g_ excited states relax radiatively to the ^2^E_g_ energy level, and then return to the ^4^A_2g_ ground state through spin-forbidden transitions from the ^2^E_g_ excited state to form the PL emission spectra^37^, as shown in Fig. 5b. The luminescence intensity of IASP-KSFM is 1.05 times that of KSFM, which indicates that the treatment of the IASP strategy enhances the luminescence intensity of the phosphors.

In order to investigate the reason for the enhanced luminescence intensity of IASP-KSFM sample, the internal quantum yield (QY_i_) and absorbance efficiency (AE) of the samples before and after IASP treatment with varying Mn-doping concentrations were examined. As listed in Table 1, the AEs of all IASP-KSFM samples are slightly lower than the KSFM samples. This can be attributed to the fact that the IASP process reduces the Mn^4+^ ions on the surface, which causes the Mn^4+^ absorption centers in the IASP-KSFM samples to be marginally smaller than those in the comparable KSFM phosphors. As the Mn^4+^ concentration is lower than 6.00 at%, the KSFM samples show high QY_i_s above 82%, while the IASP-KSFM samples exhibit higher QY_i_s exceeding 92%. However, when the Mn^4+^ concentrations are further increased from 6.00 at% to 8.00 at%, the QY_i_ of the KSFM sample decreases significantly from 82 to 65% due to the concentration quenching effect^38^. As reported by Huang et al.^39^, the energy migration probability and migration distance will increase with the increase of Mn^4+^ concentration, which enhances the possibility of being captured by the non-radiative centers^40^. As Mn^4+^-free KSF shell is formed, the surface defects are passivated and therefore the energy migration paths to surface defects are cut off by the protective shell. This reduces the non-radiative transition and increase the QY_i_s of IASP-KSFM samples. After IASP treatment, the IASP-KSFM phosphors achieved a maximum QY_i_s of 94.24% at the Mn^4+^ concentration of 6.00 at% (Fig. S4). Notably, the IASP-KSFM sample maintains a high QY_i_s of 88%, when the Mn^4+^ concentration is 8.00 at%. For typical KSFM phosphors, significant concentration quenching occurs at Mn levels greater than 4 at% and becomes progressively worse at higher concentrations of Mn. Therefore, the IASP treatment can effectively increase the critical concentration of PL quenching effect. This is important, as high Mn^4+^ concentrations are required for sufficient absorption of blue LED light in the parity-forbidden Mn^4+^ d-d transitions. The critical concentrations of KSFM phosphors for PL quenching are also much higher than KSFM phosphors treated by ion exchange-promoted surface reduction strategy (~ 6 at%) and KTFM phosphors treated by reverse cation exchange strategy (~ 7 at%). As reported by Senden^41^, at high Mn^4+^ dopant concentrations, the low PL intensities and QY_i_s are mainly originated from the increase of the probability for energy transfer to quenchers, such as surface defects.

**Table 1:** The absorption efficiency (AE) and internal quantum yields (QY_i_) of KSFM and IASP-KSFM samples with different Mn-doping concentrations.

Mn^4+^ (at%)	KSFM		IASP-KSFM	
Theoretical	QY_i_ (%)	AE (%)	QY_i_ (%)	AE (%)
2.00	93.67	48.68	92.43	44.09
4.00	86.47	50.42	91.05	45.55
6.00	82.13	53.29	94.24	46.38
8.00	64.97	57.34	88.43	52.87
10.00	54.17	60.09	77.22	53.26

To give the optimized concentration of reducing agent (H_2_KO_2_P), the PL spectra of IASP-KSFM samples treated by variant mass ratio of H_2_KO_2_P/KSFM were examined. As shown in Fig. 5c, the integrated PL intensity reaches its maximum at the mass ratio of 0.5. Further increasing the mass ratio will lead to the excessive use of the reducing agent, which causes a significant reduction of luminescent centers inside the KSFM phosphors. At low mass ratio of H_2_KO_2_P/KSFM, the reducing rate is relatively low. This might cause Mn ion residue in the KSF shell. As shown in Fig. S5, a concentration gradient of Mn can be found at the KSF/KSFM interface. Further XPS depth spectra show that, at ~ 200 nm to the surface, weak signals of Mn^3+^ 2p_3/2_ were detected in the range 650–645 eV in the KSFM treated by mass ratio of 0.3. As reported by Senden, the Mn^3+^ can be served as quenching centers in KSFM phosphors, which causes the reduction of PL intensity^41^. However, no obvious Mn signals were observed in the KSFM treated by mass ratio of 0.5. This implies that the fast reduction of Mn^4+^ is beneficial to reduce the residue of Mn at the KSF/KSFM interface. Herein quenching centers are substantially decreased. This result is consistent with the increase of the critical quenching concentrations. Such PL behavior indicates that the proposed IASP strategy can not only enhance the moisture resistant capabilities, but also improve the luminescent performance by reducing the quenchers in the prepared KSFM phosphors.

The temperature dependent PL spectra of KSFM (0.06 Mn^4+^) and IASP-KSFM (0.06 Mn^4+^) samples under 468 nm excitation were carried out to gain a deeper understanding of the basic mechanisms behind the PL enhancement in phosphors treated by the IASP method. The results are presented in Fig. 5d,e. One can find the temperature only affects the intensity of the PL peaks but not the position of the emission peaks. The integration of the luminescence intensity of the two samples is performed and the results are shown in Fig. 5f,g. As the temperature is increased to 480 K, the integrated PL intensity of KSFM (0.06 Mn^4+^) drops to 78% of its initial value measured at room temperature in Fig. 5f. In contrast, the integrated PL intensity of the IASP-KSFM (0.06 Mn^4+^) samples remains 85% at 480 K, indicating that this sample has better temperature stability (Fig. 5g). For the IASP-KSFM (0.06 Mn^4+^) samples, the integrated PL intensity reaches the maximum value of about 110.3% at around 420 K, exhibiting a significant negative thermal quenching (NTQ) effects. The NTQ effects of Mn^4+^ luminescence in the IASP-KSFM (0.06 Mn^4+^) samples can be understood by the analysis model proposed by Sadao Adachi^42^. The temperature-dependent PL intensity, *I*_PL_(*T*)_,_ of Mn^4+^-activated fluoride phosphors can be expressed as:

4 $${I_{PL} \;(T) = I_{0} \left[ {1 + \frac{2}{{\exp (hv_{s} /k_{B} T) - 1}} + 2\alpha T} \right] \times \frac{1}{{1 + \mathop \sum \nolimits_{j} a_{j} \exp \left( { - \frac{{E_{qj} }}{{k_{B} T}}} \right)}}}$$

where αT comes from the acoustic-phonon contribution,$v_{s}$ represents the effective electron–lattice interaction strength from both the ^2^*E*_g_(*ν*_s,0_) and ^4^*T*_2g_(*ν*_ex_), $E_{qj}$ represents the thermal quenching energy, and $a_{j}$ represents the strength parameters of the corresponding quenching pathway $j$. In this study, we consider two different thermal quenching path ways of j = 1 and 2, where the quenching pathways of j = 1 corresponds to non-radiative recombination processes via bulk and surface defect states, while the thermal quenching pathway of j = 2 is dependent on thermally assisted non-radiative recombination processes^43^ shown in Fig. S6.

Figure 5f,g show the experimental I_PL_(T) data for both KSFM (0.06 Mn^4+^)and IASP-KSFM (0.06 Mn^4+^) samples respectively. The red solid lines represent the best-fit results calculated using Eq. (4). An excellent agreement between the experimental and theoretical curves for both samples can be achieved over the entire temperature range. It can be seen the fit-determined parameters: ${\text{h}}\nu_{s}$, $a_{2}$,$E_{qj}$ for both samples are basically the same, while $a_{1}$ for IASP-KSFM (0.06 Mn^4+^) sample is much smaller than that of KSFM (0.06 Mn^4+^) phosphor. As reported previously^42^, the NTQ effect is mainly influenced by the thermal quenching pathways of j = 1, which is strongly dependent on the non-radiative recombination center densities. In this study, the KSFM (0.06 Mn^4+^) and IASP-KSFM (0.06 Mn^4+^) samples are fabricated by the same co-deposition process. For both samples, the bulk defects center densities are basically the same. Herein, the variations of $a_{1}$ are mainly originated from the recombination centers densities at the surface of phosphors. Furthermore, the decrease of $a_{1}$ proves that the IASP process can substantially passive the surface and reduce the densities of surface recombination centers in KSFM phosphors.

The results of PL decay investigations are presented in Fig. 5h,i. The entire decay curve can be well-fitted to a second-order exponential decay model by the following equation^44^:

5 $${{\text{I}}(t) = A_{1} e^{{ - t/\tau_{1} }} + A_{2} e^{{ - t/\tau_{2} }} }$$

where I is the luminescence intensity, $A_{1}$ and $A_{2}$ are constants, $t$ is the decay time, and $\tau_{1}$ and $\tau_{2}$ are the lifetimes for the exponential components. Also, the average lifetime constant (τ) can be calculated as

6 $${\tau_{ave} = \left( {A_{1} \tau_{1}^{2} + A_{2} \tau_{2}^{2} } \right)/\left( {A_{1} \tau_{1} + A_{2} \tau_{2} } \right)}$$

From Eq. (5), the decays of the excited electrons in KSFM (0.06 Mn^4+^) phosphor is mainly originated from a slower component corresponding to the radiative way and a fast component corresponding to non-radiative process via crystalline defect states or thermally assisted recombination. By Eq. (6), one can obtain the average lifetimes, 7.84 and 8.06 ms for KSFM (0.06 Mn^4+^) and IASP-KSFM (0.06 Mn^4+^), respectively. The calculated lifetimes of Mn^4+^ are in a microsecond range, indicating the forbidden character of the intra-d-shell transitions in Mn^4+^ ions. Notably, it can be seen the fit-determined parameter $A_{1}$ for IASP-KSFM (0.06 Mn^4+^) is much smaller than KSFM (0.06 Mn^4+^), resulting in a longer average lifetime in this type of sample. As shown in Fig. 5f, the non-radiative recombination in KSFM (0.06 Mn^4+^) phosphor arises mainly from thermally assisted non-radiative process and defects assisted non-radiative process. From Eq. (4), the thermal quenching energy corresponding to thermally assisted non-radiative process is 1 eV, which is much higher than phonon vibration energy (~ 65 meV for $hv_{s}$, at room temperature). This implies that the possibility of luminescence decay through thermally assisted non-radiative recombination processes is low and herein the variation of the coefficient $A_{1}$ is mainly dependent on the change of non-radiative decays via crystalline defect states. Consequently, the longer PL decay time observed in IASP-KSFM (0.06 Mn^4+^) samples can be attributed to the effective passivation of surface defects, which is consistent with the experimental results of NTQ effects,

### Long-term stability of LED devices

**Figure 6:**
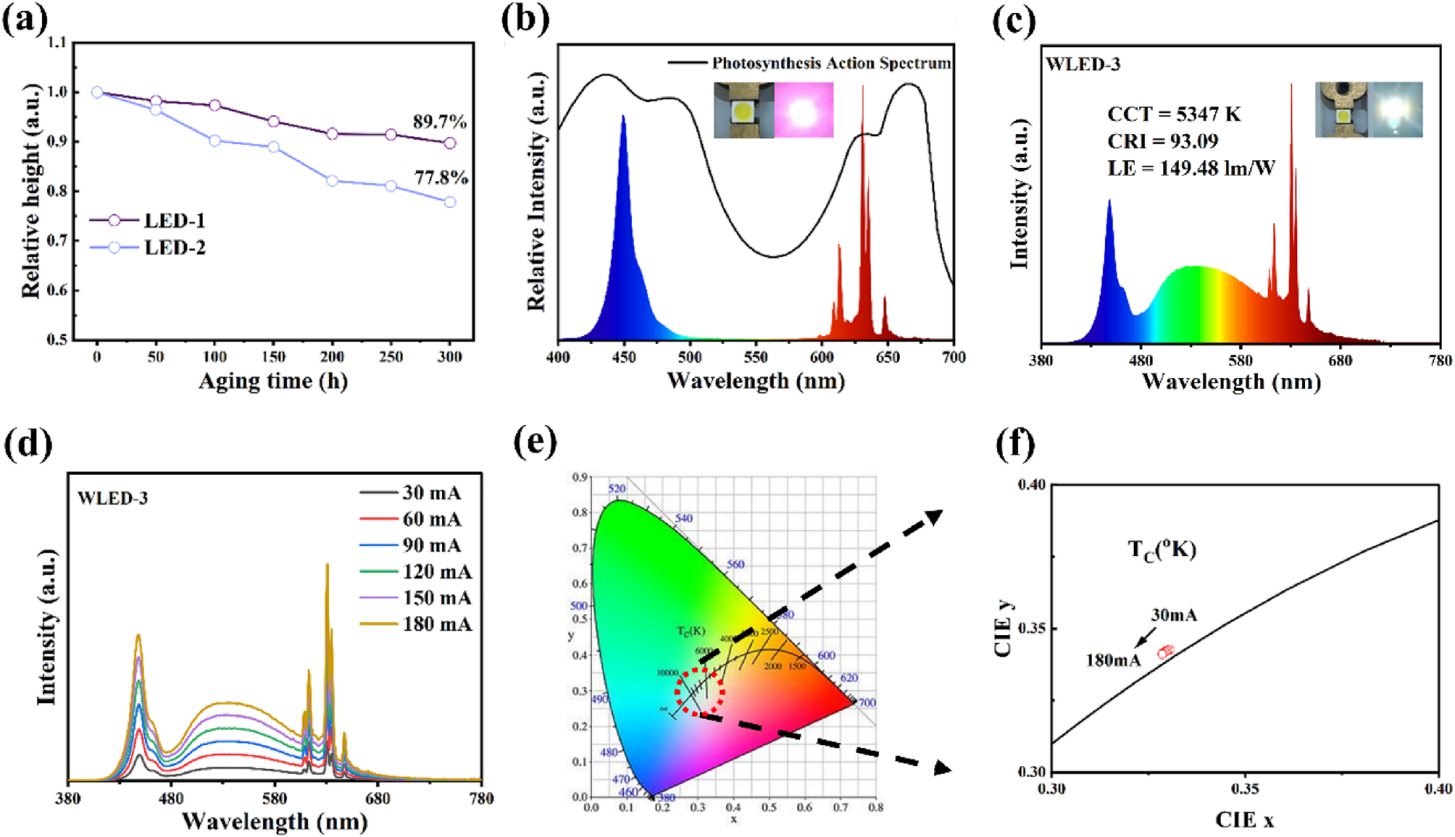
(**a**) The variation of EL intensities at 632 nm of (**a**) the LED (1 and 2) with the aging time under HT (85 °C) and HH (85%) condition and the driven current of 60 mA. (**b**) EL spectra of LED-1 with the driven current of 60 mA, the inset shows the lighted devices. The black solid line represents the photosynthesis action spectrum. (**c**) EL spectra of WLED-3 with the driven current of 60 mA. The insets show photographs of unlighted and lighted devices. (**d**) EL spectra of WLED-3 with the different driven currents. (**e**) CIE chromaticity coordinates of WLED-3 driven at different currents from 30 to 180 mA. (**f**) Enlarged image of the selected area in (**e**).

The aging tests were performed to evaluate the stability of the prepared red phosphors in a real working environment in an HT (85 °C) and HH (85%) environment. LED-1 and LED-2 were fabricated by mixing IASP-KSFM (0.06 Mn^4+^) red phosphor and KSFM (0.06 Mn^4+^) with epoxy resin. Both LEDs were excited under a blue LED (λ_max_ = 450 nm) driven by 60 mA currents. Figure 6a shows the variation of electroluminescence (EL) peak intensities of LED devices at 632 nm with the aging time by normalizing the emission peak of the chip (450 nm). One can find the EL peak intensity of LED-1 maintained approximately 89.7% of its initial value after 300 h of aging, which decreases more slowly than that of LED-2. This suggests that the IASP treatment significantly improves the long-term aging resistance of KSFM (0.06 Mn^4+^). Meanwhile, at the optimized package conditions, the spectrum of LED-1 falls within the spectrum of the photosynthesis action spectrum (Fig. 6b), which shows the potential application aspects of the IASP-KSFM (0.06 Mn^4+^) phosphor in the red component of the stable supplementary lighting system for plant growth. Next, we packaged WLED-3 by mixing the IASP-KSFM (0.06 Mn^4+^) red phosphor and commercial green phosphor (β-SiAlON: Eu^2+^) coated on a blue chip. Figure 6c,d show the EL spectra of WLED-3 under a blue LED driven by various currents. The related parameters, such as CCT and CRI, are listed in Table S2. As shown in Fig. 6e,f, the chromaticity coordinates of WLED-3 driven at different currents from 30 to 180 mA were assigned to CIE 1931 color spaces, and all points lie near the black tracks. The WLED-3 driven by 60 mA exhibits CIE chromaticity coordinates of (0.3299, 0.3425), a CCT of 5347 K, a high general color rendering index (CRI) of 93.09, a color rendering index of saturated red (R9) of 86.94, and a luminous efficiency (LE) of 149.48 lm/W. The chromaticity shift (ΔE) is an important parameter to evaluate the luminescence stability of phosphors. WLED-3 of ΔE at 30 mA and 180 mA is calculated by the Eq. (S2). It can be found from the calculation results that the ΔE of WLED-3 from 30 to 180 mA is only 2.19 × 10^−3^, which indicates the IASP-KSFM (0.06 Mn^4+^) has a high color stability. This implies the potential of IASP-KSFM (0.06 Mn^4+^) as a promising candidate for long-term stable WLEDs.

## Conclusions

In summary, we demonstrated a facile and environmentally friendly IASP surface treatment strategy to prepare red emitting Mn^4+^-activated fluoride phosphors. KSFM (0.06 Mn^4+^) phosphor was treated with H_2_KO_2_P in a mixed solution of saturated HF-KSF. A thick and clean Mn^4+^-free shell layer was constructed on its surface to improve water resistance. The stability test of fluoride in water at room temperature and boiled water showed that the IASP-treated KSFM (0.06 Mn^4+^) phosphor has an excellent water resistant property. Meanwhile, the fitting results of NTQ and PL decay proved that the IASP process can substantially passivate the surface and reduce the densities of surface recombination centers. The maximum QY_i_ of the IASP-KSFM (0.06 Mn^4+^) phosphor is 94.24%. The aging results of LED devices in HT (85 °C) and HH (85%) environments further confirmed that the color stability of the IASP-KSFM (0.06 Mn^4+^) phosphor significantly improved. A white LED encapsulated by combining IASP-KSFM (0.06 Mn^4+^) with a common blue GaN chip and commercial green β-SiAlON: Eu^2+^ phosphors realized a high CRI of 93.09 at CIE color coordinates (0.3299, 0.3425) with a CCT of 5347 K and high LE of 149.48 lm/W. The excellent moisture resistance and luminescence efficiency of the IASP-KSFM (0.06 Mn^4+^) phosphor demonstrated that it has the potential use in plant supplementary lighting systems and WLEDs.

### Supplementary Information

Supplementary Information.

## Data Availability

Data is provided within the manuscript or supplementary information files.

## References

[1] Oh JH, Eo YJ, Yoon HC (2016). Evaluation of new color metrics: Guidelines for developing narrow-band red phosphors for WLEDs. J. Mater. Chem. C.

[2] Ye S, Xiao F, Pan YX (2010). Phosphors in phosphor-converted white light-emitting diodes: Recent advances in materials, techniques and properties. Mater. Sci. Eng. R. Rep..

[3] Li G, Tian Y, Zhao Y (2015). Recent progress in luminescence tuning of Ce^3+^ and Eu^2+^-activated phosphors for pc-WLEDs. Chem. Soc. Rev..

[4] Nag A, Panigrahi K (2022). Challenges and strategies to design phosphors for future white light emitting diodes. J. Phys. Chem..

[5] Chen D, Zhou Y, Zhong J (2016). A review on Mn^4+^ activators in solids for warm white light-emitting diodes. RSC Adv..

[6] Zhong J, Chen D, Zhao W (2015). Garnet-based Li_6_CaLa_2_Sb_2_O_12_:Eu^3+^ red phosphors: A potential color-converting material for warm white light-emitting diodes. J. Mater. Chem. C.

[7] Lin CC, Meijerink A, Liu RS (2016). Critical red components for next-generation white LEDs. J. Phys. Chem. Lett..

[8] Shakhno A, Zorenko T, Witkiewicz-Łukaszek S (2023). Ce^3+^ doped Al_2_O_3_-YAG eutectic as an efficient light converter for white LEDs. Materials.

[9] Xi L, Pan Y (2017). Tailored photoluminescence properties of a red phosphor BaSnF_6_:Mn^4+^ synthesized from Sn metal at room temperature and its formation mechanism. Mater. Res. Bull..

[10] Jang MK, Cho YS, Huh YD (2020). Preparation of red-emitting BaSiF_6_:Mn^4+^ phosphors for three-band white LEDs. Opt. Mater..

[11] Subhoni M, Zafari U, Srivastava AM (2021). First-principles investigations of geometrical and electronic structures of Mn^4+^ doped A_2_SiF_6_ (A = K, Rb, Cs) red phosphors. Opt. Mater..

[12] Ming H, Liu S, Liu L (2018). Highly regular, uniform K_3_ScF_6_:Mn^4+^ phosphors: Facile synthesis, microstructures, photoluminescence properties, and application in light-emitting diode devices. ACS Appl. Mater. Interfaces.

[13] Deng TT, Song EH, Zhou YY (2017). Stable narrowband red phosphor K_3_GaF_6_:Mn^4+^ derived from hydrous K_2_GaF_5_(H_2_O) and K_2_MnF_6_. J. Mater. Chem. C.

[14] Pust P, Weiler V, Hecht C (2014). Narrow-band red-emitting Sr [LiAl_3_N_4_]:Eu^2+^ as a next-generation LED-phosphor material. Nat. Mater..

[15] Zhang Y, Zhang Z, Liu X (2020). A high quantum efficiency CaAlSiN_3_:Eu^2+^ phosphor-in-glass with excellent optical performance for white light-emitting diodes and blue laser diodes. Chem. Eng. J..

[16] Kasa R, Adachi S (2012). Red and deep red emissions from cubic K_2_SiF_6_:Mn^4+^ and hexagonal K_2_MnF_6_ synthesized in HF/KMnO_4_/KHF_2_/Si solutions. J. Electrochem. Soc..

[17] Verstraete R, Sijbom HF, Korthout K (2017). K_2_MnF_6_ as a precursor for saturated red fluoride phosphors: The struggle for structural stability. J. Mater. Chem. C.

[18] Liu Z, Liu Y, Pan C (2020). K_2_MnF_6_/KHF_2_ red phosphor synthesis by a low temperature way for high color rendering index white light emitting diodes. Ferroelectrics.

[19] Yang J, Luo P, Wan P (2022). Surface engineered environment-stable red-emitting fluorides for white light emitting diodes. Ceram. Int..

[20] Arunkumar P, Kim YH, Kim HJ (2017). Hydrophobic organic skin as a protective shield for moisture-sensitive phosphor-based optoelectronic devices. ACS Appl. Mater. Interfaces.

[21] Nguyen HD, Lin CC, Liu RS (2015). Waterproof alkyl phosphate coated fluoride phosphors for optoelectronic materials. Angew. Chem..

[22] Zhou YY, Song EH, Deng TT (2018). Waterproof narrow-band fluoride red phosphor K_2_TiF_6_:Mn^4+^ via facile superhydrophobic surface modification. ACS Appl. Mater. Interfaces.

[23] Dong Q, Guo C, He L (2019). Improving the moisture resistance and luminescent properties of K_2_TiF_6_:Mn^4+^ by coating with CaF_2_. Mater. Res. Bull..

[24] Verstraete R, Rampelberg G, Rijckaert H (2019). Stabilizing fluoride phosphors: Surface modification by atomic layer deposition. Chem. Mater..

[25] Zhao Y, Guan Q, Wang H (2022). Waterproof surface passivation of K_2_GeF_6_:Mn^4+^ by a dense Al_2_O_3_ layer via atomic layer deposition. J. Mater. Chem. C.

[26] Suehiro T, Xie RJ, Hirosaki N (2014). Gas-reduction–nitridation synthesis of CaAlSiN_3_:Eu^2+^ fine powder phosphors for solid-state lighting. Ind. Eng. Chem. Res..

[27] Liu Y, Li Y, Feng Y (2020). Effects of graphene quantum dots coating on the luminescence properties of K_2_SiF_6_:Mn^4+^ red-emitting phosphors. J. Mater. Sci. Mater. Electron..

[28] Qiang J, Wang L, Wang T (2022). Improvement of the luminescent thermal stability and water resistance of K_2_SiF_6_:Mn^4+^ by surface passivation. Ceram. Int..

[29] Huang L, Liu Y, Si S (2018). A new reductive dl-mandelic acid loading approach for moisture-stable Mn^4+^ doped fluorides. Chem. Commun..

[30] Yu H, Wang B, Bu X (2020). A facile in situ surface-coating passivation strategy for improving the moisture resistance of Mn^4+^-activated fluoride red phosphor. Ceram. Int..

[31] Zhou Y, Song E, Deng T (2019). Surface passivation toward highly stable Mn^4+^-activated red-emitting fluoride phosphors and enhanced photostability for white LEDs. Adv. Mater. Interfaces.

[32] Ruan H, Wang T, Wang L (2023). Ion exchange-promoted surface reduction strategy: For improving the water-resistance of K_2_SiF_6_:Mn^4+^ red phosphors. Ceram. Int..

[33] Liu YX, Hu JX, Ju LC (2020). Hydrophobic surface modification toward highly stable K_2_SiF_6_:Mn^4+^ phosphor for white light-emitting diodes. Ceram. Int..

[34] Pan X, Wu D, Liu S (2023). A novel thiourea loading strategy for improving the moisture resistance of K_2_SiF_6_:Mn^4+^ red phosphors. Mater. Res. Bull..

[35] Wan P, Liang Z, Luo P (2021). Reconstruction of Mn^4+^-free shell achieving highly stable red-emitting fluoride phosphors for light-emitting diodes. Chem. Eng. J..

[36] Adachi S, Takahashi T (2008). Direct synthesis and properties of K_2_SiF_6_:Mn^4+^ phosphor by wet chemical etching of Si wafer. J. Appl. Phys..

[37] Yu Y, Wang T, Deng D (2022). Enhancement of the luminescent thermal stability and water resistance of K_2_SiF_6_:Mn^4+^, Na^+^ by double coating of GQDs and K_2_SiF_6_. J. Alloys Compd..

[38] Zhu H, Lin CC, Luo W (2014). Highly efficient non-rare-earth red emitting phosphor for warm white light-emitting diodes. Nat. Commun..

[39] Huang D, Zhu H, Deng Z (2019). Moistureresistant Mn^4+^-doped core–shellstructured fluoride red phosphor exhibiting high luminous efficacy for warm white lightemitting diodes. Angew. Chem. Int. Ed..

[40] Dexter DL, Schulman JH (1954). Theory of concentration quenching in inorganic phosphors. J. Chem. Phys..

[41] Senden T, van Dijk-Moes RJA, Meijerink A (2018). Quenching of the red Mn^4+^ luminescence in Mn^4+^ doped fluoride LED phosphors. Light Sci. Appl..

[42] Adachi S (2022). negative thermal quenching of Mn^4+^ luminescence in fluoride phosphors: Effects of the ^4^A_2g_ → ^4^T_2g_ excitation transitions and normal thermal quenching. ECS J. Solid State Sci. Technol..

[43] Chang C, Ye W, Zuo C (2023). Highly moisture-stable and enhanced luminescence-efficient Mn^4+^-activated red-emitting fluoride phosphors via a bi-hydrogen-bond organic coating. ACS Sustain. Chem. Eng..

[44] Yong ZJ, Guo SQ, Ma JP (2018). Doping-enhanced short-range order of perovskite nanocrystals for near-unity violet luminescence quantum yield. J. Am. Chem. Soc..

