# Supplementary material for: Ion exchange-assisted surface passivation toward highly stable red-emitting fluoride phosphors for light-emitting diodes
Source: Sci Rep. 2024 Jun 26;14:14695. doi: 10.1038/s41598-024-65169-z (PMC11208521; doi:10.1038/s41598-024-65169-z)
Supplement: Supplementary file 1 — Supplementary Information. [file 41598_2024_65169_MOESM1_ESM.docx]

**Supporting Information**

Ion exchange-assisted surface passivation toward highly stable red-emitting fluoride phosphors for light-emitting diodes

# 1. Experimental procedures

## 1.1 Chemicals and Materials

Potassium permanganate (KMnO_4_, 99.5%), potassium hydrogen fluoride (KHF_2_, 99%), HF solution(49 wt%, ≥ 99.9%)，Methyl alcohol (CH_3_OH, 99.5%), ethyl alcohol (CH_3_CH_2_OH, 99%), hydrogen peroxide solution (H_2_O_2_，30 wt%), Hexafluorosilicic acid (H_2_SiF_6_, 30~32%), Potassium hypophosphite (H_2_KO_2_P, 99%) and Potassium fluorosilicate (K_2_SiF_6_, 99%) were purchased from Macklin Reagent. All the chemicals were used directly without further purification. The K_2_MnF_6_ crystals were prepared based on Bode's method. ln a typical synthetic process, 10 g of KHF_2_ and 0.5 g of KMnO_4_ were first dissolved in 30 ml of HF solution (49 wt%) which was kept in ice bath and stirred for 60 min. Next 0.32 ml of 30% H_2_O_2_ solution was added dropwise to the solution slowly using a dropper. At the end of the reaction, the color of the solution turned from deep-purple to golden yellow together with the precipitation of K_2_MnF_6_ powders, which were collected instantly by fltration, followed by washing with HF and ethanol several times and drying at 70°C for 3 h in an vacuum oven.

## 1.2 Synthesis

### 1.2.1 Synthesis of K_2_SiF_6_ : *x*Mn^4+^ (KSFM : *x*Mn^4+^)

Firstly, 8 mmol of H_2_SiF_6_ solution and 20 ml HF (49%) solution were mixed and stirred for 15 minutes to obtain solution H_2_SiF_6_-HF solution. Subsequently,0.5 mmol of K_2_MnF_6_ was dissolved into the H_2_SiF_6_-HF solution stirring for 30 min. At the same time,24 mmol of KHF_2_ was added into10 ml of 49% HF solution to prepare KHF_2_-HF solution.Finally,the KHF_2_-HF solution was added dropwise to H_2_SiF_6_-HF solution to precipitate the yellow K_2_SiF_6_:0.06 Mn^4+^ powers. The as-precipitated yellow powders were collected by filtration, washed with ethanol several times, and oven-dried at 60°C for 4 h. The K_2_SiF_6_ of other Mn^4+^ doping concentrations can be obtained by changing the amount of K_2_MnF_6_ added.

### 1.2.2 Treatment of KSFM(0.06 Mn^4+^) by surface passivation strategy (SP-KSFM)

1 g of H_2_KO_2_P power was dissolved in 10 ml deionized water to obtain H_2_KO_2_P solution.Then,2 g of K_2_SiF_6_:0.06 Mn^4+^ was added into H_2_KO_2_P solution and stirred for 30 min at room temperature.Finally, the product was filtered, collected and washed with methyl alcohol several times and dried at 70℃ for 2 h to get the SP-KSFM phosphors.

### 1.2.3 Treatment of KSFM(0.06 Mn^4+^) by Ion exchange-assisted surface passivation strategy (IASP-KSFM)

The typical treatment steps of IASP are as follows: Firstly, 2 g of K_2_SiF_6_:0.06 Mn^4+^ was dissolved in 10 ml of saturated K_2_SiF_6_ solution and stirred for 30 min to proceed cation exchange adequately. Secondly, the H_2_KO_2_P solution is dropped slowly and stirring continued for 20 min. Finally, the obtained was centrifuged, washed with methyl alcohol several times and placed in oven at 70 ℃ for 2 h to dry.

## 1.3 WLED package and performance measurement

LED-1 and LED-2 were package by fluoride power and epoxy resin uniformly and then coated on the blue-chip (~450 nm). They were put in an aging box (LRHS-101-LH) for 300 h at high temperature (85℃) and high humidity (85%). White LED were packaged by combining blue diode chips (~450 nm), aluminate green phosphor (β-SiAlON:Eu^2+^), KSFM or IASP-KSFM red phosphor. The phosphors were mixed with epoxy resin thoroughly and the mixture was used to coat the surface of the blue chips. After mixing uniformly, the mixtures were coated on the blue chips and dried at 130 ℃ for 10 hours to obtain WLED-3 devices.The photoelectric properties of the fabricated devices were measured by LED optoelectronic analyzer. The white LED were operated under a voltage of 3.0 V with various drive currents ranging from 30 to 180 mA.

## 1.4 Material characterizations

The X-ray powder diffraction (XRD) patterns of the samples were results from Cu Kα radiation (λ=1.5406 Å) of the X-ray diffractometer (Rigaku Smart Lab 9 kw, Rigaku, Japan). The microscopic morphologies of the samples were investigated with a field-emission scanning electron microscope (SEM, ZEISS Sigma 300, Carl Zeiss AG, Germany) equipped with an energy dispersive X-ray spectroscopy (EDS) analyzer. The photoluminescence excitation(PLE), photoluminescence(PL) spectra and luminescence decay curve were detected using an Hitachi F-4700 fluorescence spectrometer equipped with a 450 W Xenon lamp as the light source. For internal quantum yield (QY_i_) and absorption efficiency (AE) measurement, the samples were measured using an absolute QY_i_ spectrometer (Edinburgh FLS-1000, UK) under 462 nm excitation. The temperature-dependent PL emission spectra were analyzed by an FluoroMax Plus spectrometer. The elements of the samples and their chemical bonding states were characterized using X-ray photoelectron spectroscopy (XPS, Thermo ESCALAB 250Xi, Thermo Fisher Scientific, US) with an Al Kα source.

# Theoretical caculation

## 2.1 The internal quantum yield

The internal quantum yield (QYi) of samples is calculated based on Eq. (S1)

$$\begin{aligned} QY_{i}=\frac{\int L_{emission}}{\int E_{black}-\int E_{sample}}\#\left( S1 \right) \end{aligned}$$

Where *L*_emission_ is the emission spectrum of the sample, *E*_blank_ and *E*_sample_ refer to the excitation light spectra without and with samples in the integrating sphere, respectively.

## 2.2 Chromaticity shift

The chromaticity shift (*ΔE*) at different driven currents is calculated using Eq. (S2)

$$\begin{aligned} \Delta E=\sqrt{(u_{2}^{'}-u_{1}^{'})^{2}+(v_{2}^{'}-v_{1}^{'})^{2}+(w_{2}^{'}-w_{1}^{'})^{2}}\#\left( S2 \right) \end{aligned}$$

Where *u’* = *4x/(3−2x+12y)*, *v’* = *9y/(3−2x+12y)* and *w’* = *1−u’−v’*. *x* and *y* are the chromaticity coordinates in CIE1931 color space, *u'* and *v'* are the chromaticity coordinates in the uniform color space, and *1* and *2* are the chromaticity shift at 30 mA and a given driven currents, respectively.

# Results and discussion


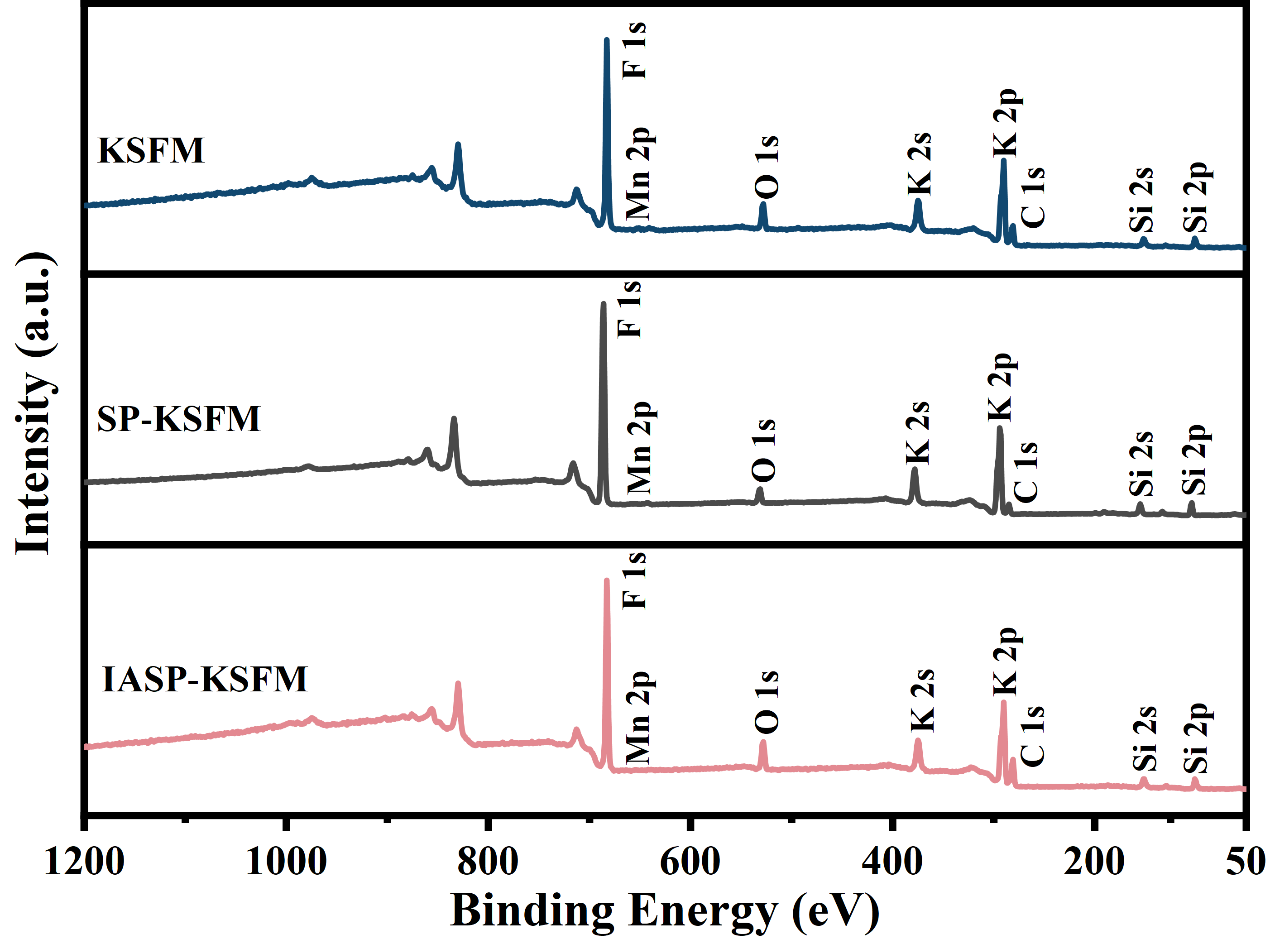


**Fig. S1**. Complete XPS spectra of KSFM , SP-KSFM and IASP-KSFM samples.


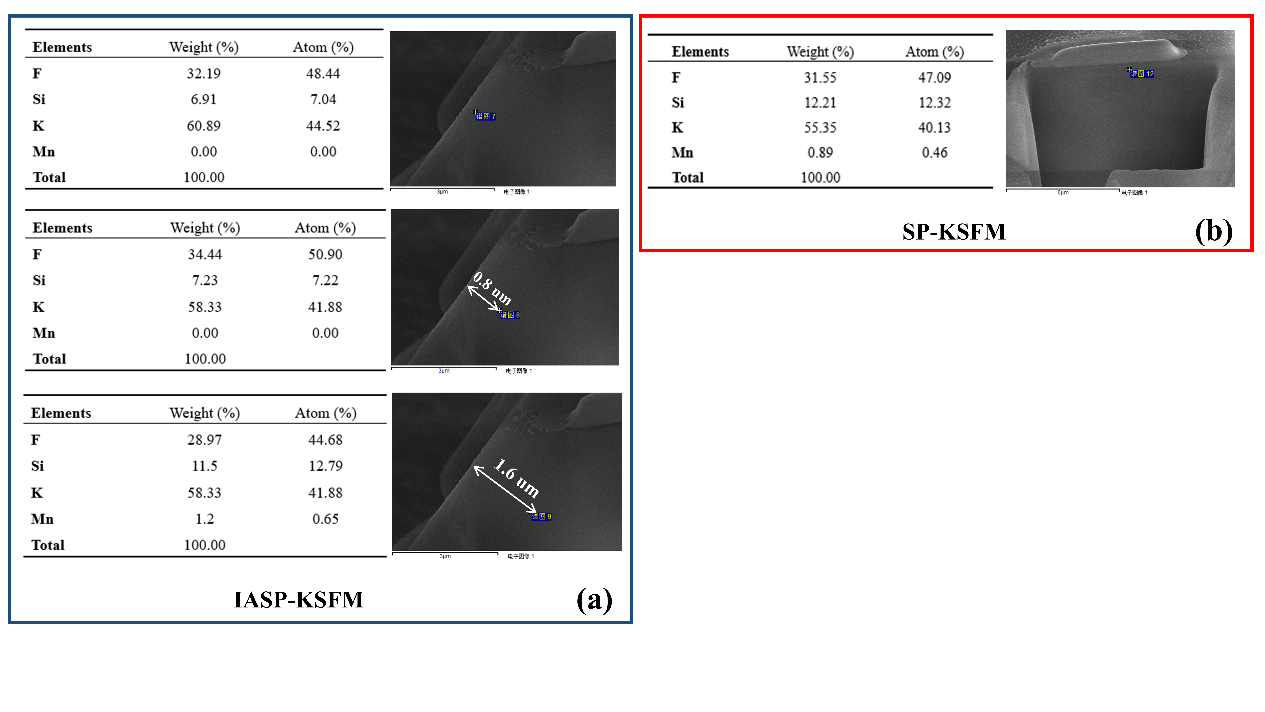


(c)


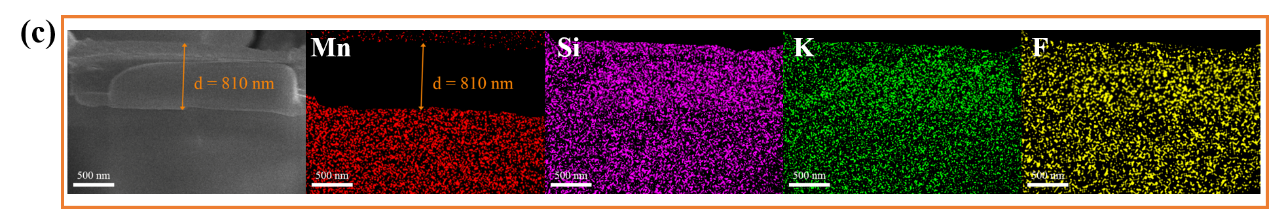


**Fig. S2.** FIB test datas and images of IASP-KSFM and SP-KSFM, (a) IASP-KSFM, (b) SP-KSFM (c)SEM cross-sectional images of IASP-KSFM sample. Element mapping of Mn, Si, K, and F performed on the cross section by EDS.


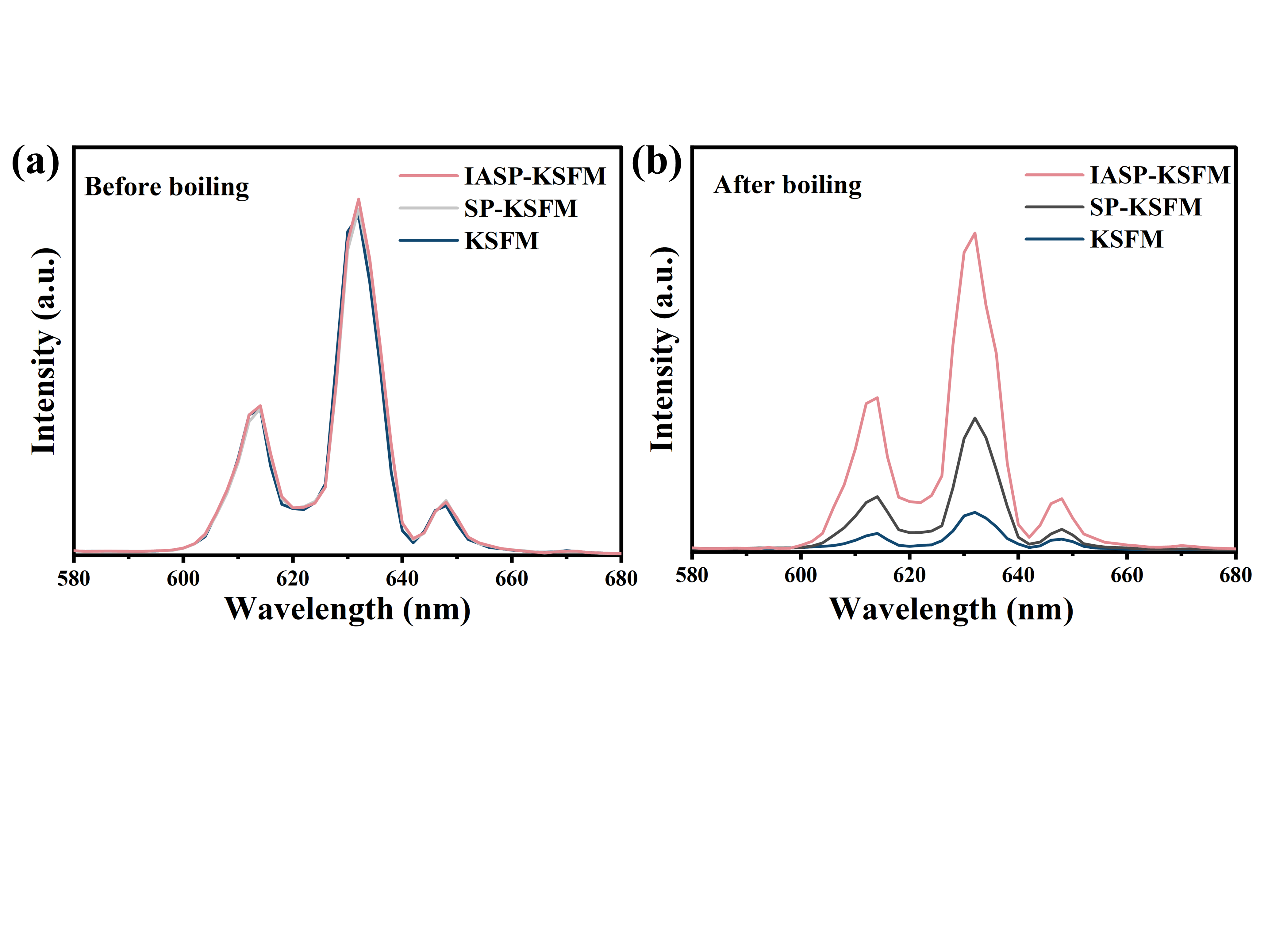


**Fig. S3.** Luminescence properties of KSFM, SP-KSFM and IASP-KSFM. (a) Before boiling, (b) After boiling.


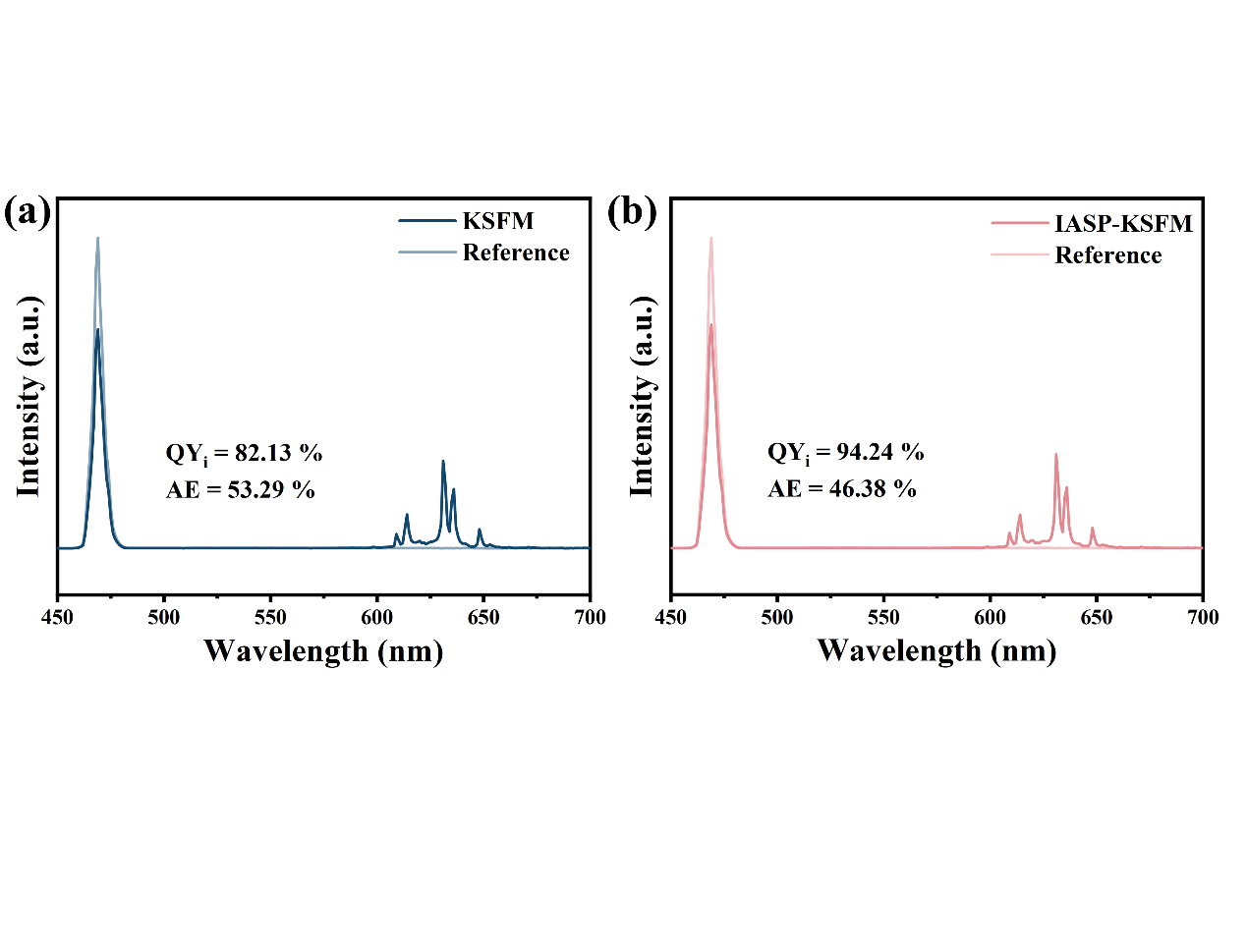


**Fig. S4.** (a, b) Internal quantum yield (QY_i_) and absorption efficiency (AE) for the samples are measured by integrating, (a) KSFM and (b) IASP-KSFM.


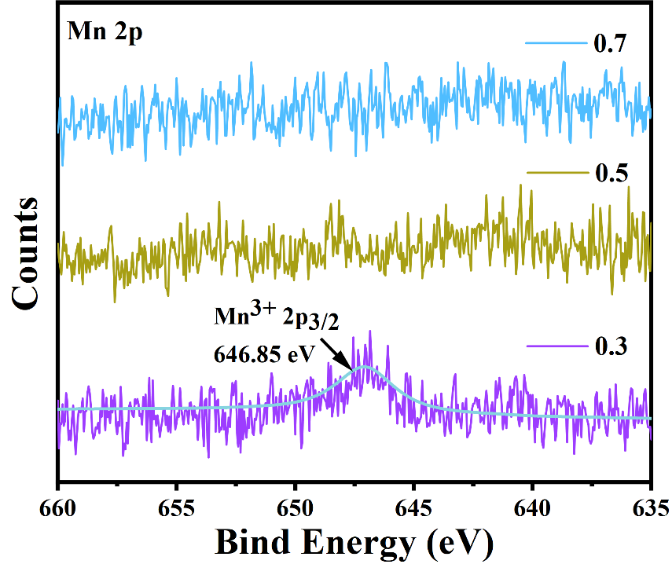


**Fig. S5.** High-resolution XPS spectra of Mn2p at different H_2_KO_2_P/KSFM specific weights (0.3, 0.5, 0.7).


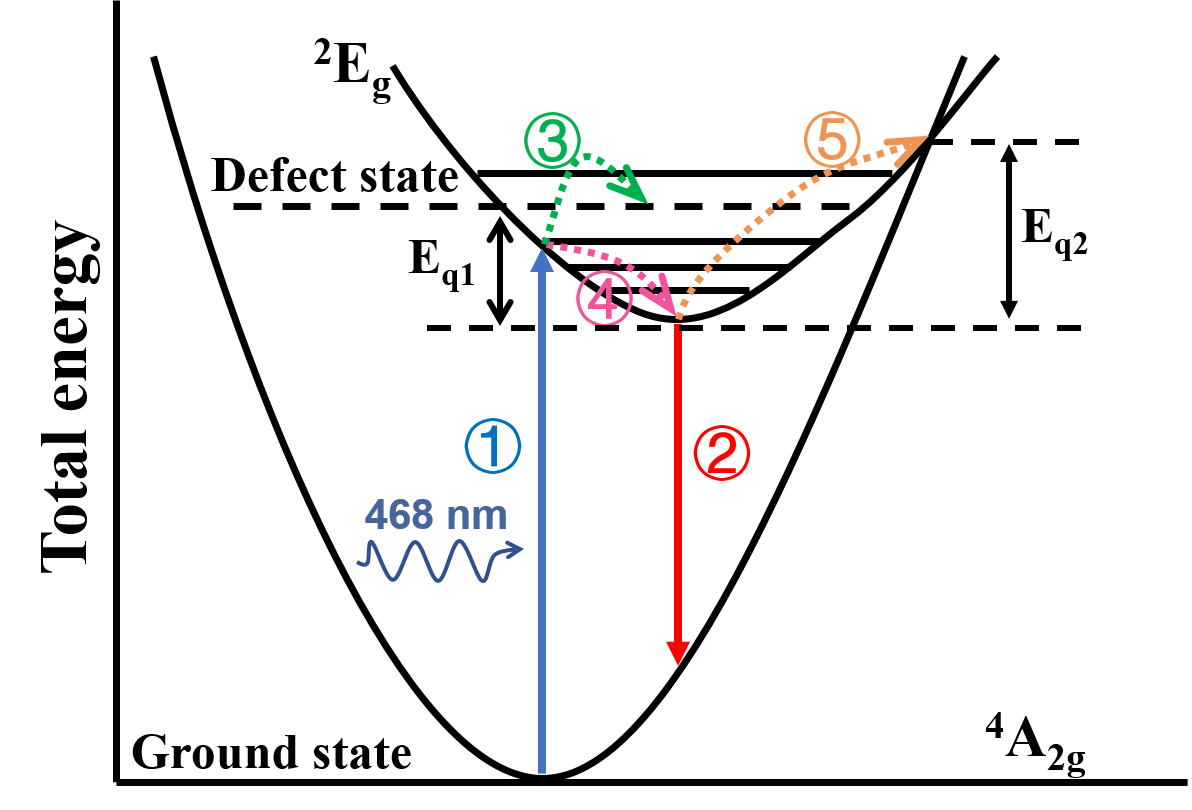


**Fig. S6.** Illustration diagrams for luminescent quenching model under configuration coordinates. Progression ① represents the jump of the electrons of Mn^4+^ from the ground state to the excited state under blue light excitation. Progression ② represents the excited electrons return to the ground state through process, realizing red luminescence. Progression ③ represents non-radiative recombination processes via bulk and surface defect states. Progression ⑤ represents thermally assisted non-radiative recombination.

**Table S1.** Refined lattice parameters of samples.

| Samples | a=b=c/Å | α=β=γ/° | V/ Å^3^ | Space group |
| --- | --- | --- | --- | --- |
| KSFM | 8.1775 | 90 | 546.84 | Fm-3m |
| SP-KSFM | 8.1604 | 90 | 543.42 | Fm-3m |
| IASP-KSFM | 8.1432 | 90 | 539.99 | Fm-3m |
| PDF#75-0694 | 8.1340 | 90 | 538.2 | Fm-3m |

**Table S2.**Optical properties parameters of the WLED-3 under various currents.

| Current(mA) | CCT(K) | CIE color coordinate | | CRI(Ra) | R9 | LE(lm/W) |
| --- | --- | --- | --- | --- | --- | --- |
|  |  | x | y |  |  |  |
| 30 | 5342 | 0.33046 | 0.34268 | 93.23 | 87.45 | 152.09 |
| 60 | 5347 | 0.32995 | 0.34248 | 93.09 | 86.94 | 149.48 |
| 90 | 5355 | 0.32955 | 0.34225 | 92.95 | 86.57 | 145.58 |
| 120 | 5373 | 0.3292 | 0.34181 | 92.88 | 86.30 | 142.23 |
| 150 | 5379 | 0.32868 | 0.34145 | 92.71 | 86.08 | 139.19 |
| 180 | 5384 | 0.32855 | 0.34117 | 92.68 | 85.89 | 136.17 |

**Table S3.**QY_i_ and relative PL intensities after soaking in water for different time of the treated fluorides.

| Treatment type | Water resistance  (relative to initial intensity) | QYi | Reference |
| --- | --- | --- | --- |
| H_2_C_2_O_4_ passivated K_2_GeF_6_:Mn^4+^ | 300 min, 95.8% | 62.4% | [8] |
| Na_2_SO_3_ passivated K_2_SiF_6_:Mn^4+^ | 300 min, 75.8% | 98% | [9] |
| Glyoxylic acid passivated K_2_SiF_6_:Mn^4+^ | 360 h, 97.7% | 96.37% | [10] |
| K_2_SiF_6_:Mn^4+^@C | 8 h, 83% |  | [11] |
| K_2_SiF_6_:Mn^4+^@ K_2_SiF_6_ | 6 h, 76% | 59% | [12] |
| Oleic acid passivated K_2_SiF_6_:Mn^4+^ | 4 h, 44% | 68.1% | [13] |
| Oxalic acid passivated K_2_SiF_6_:Mn^4+^ | 5 h, 62.3% |  | [14] |
| Pyruvic acid passivated K_2_SiF_6_:Mn^4+^ | 360 h, 88.5% | 94.18% | [15] |
| Thiourea loaded K_2_SiF_6_:Mn^4+^ | 168 h, 93.5% | 86.1% | [16] |
| Ascorbic acid-KSFM-RSRC | 360 h, 97% | 96.68% | [17] |
| Na_2_NO_2_-IESR-KSFM | 8 h 90.2% | 88.86% | [18] |
| KSFM@OAm-3x | 6 h, 83.8% | 94.2% | [19] |
| KH_2_PO_2_-IASP-KSFM | 7 h, 97 % | 94.24% | This work |
|  | After boiling, 82% |  |  |

# Extra material

Video of boiling text for three samples: from left to right are KSFM, SP-KSFM, and IASP-KSFM

# References

1. L. Wang, D. Deng, J. Qiang, J. Lei, T. Wang, Y. Li, S. Liao, Y. Huang, Mn-Activated Fluoride Phosphors Modified by Surfactant with Outstanding Water Resistance and Luminescent Thermal Properties, Inorg. Chem. 62 (2023) 4157-4169.
2. H. Bode, H. Jenssen, F. Bandte, Über eine neue darstellung des kalium hexafluoromanganats (iv), Angew. Chem. 65 (1953) 304.
3. J. Qiang, L. Wang, T. Wang, Y. Yu, D. Deng, C. Wu, S. Liao, S. Li, Improvement of the luminescent thermal stability and water resistance of K_2_SiF_6_:Mn^4+^ by surface passivation, Ceram. Int. 48 (2022) 17253-17260.
4. S. Adachi, Review-Mn^4+^-activated red and deep red-emitting phosphors, ECS J. Solid State Sci. Technol. 9 (2020), 016001.
5. Ruan H, Wang T, Wang L, et al. Ion exchange-promoted surface reduction strategy: For improving the water-resistance of K2SiF6: Mn4+ red phosphors[J]. Ceramics International, 2023, 49(22): 35165-35174.
6. Chang C, Ye W, Zuo C, et al. Highly Moisture-Stable and Enhanced Luminescence-Efficient Mn^4+^-Activated Red-Emitting Fluoride Phosphors via a Bi-hydrogen-Bond Organic Coating[J]. ACS Sustainable Chemistry & Engineering, 2023, 11(44): 15887-15897.
7. Wan P, Liang Z, Luo P, et al. Reconstruction of Mn^4+^-free shell achieving highly stable red-emitting fluoride phosphors for light-emitting diodes[J]. Chemical Engineering Journal, 2021, 426: 131350.
8. Yu H, Wang B, Bu X, et al. A facile in situ surface-coating passivation strategy for improving the moisture resistance of Mn^4+^-activated fluoride red phosphor[J]. Ceramics International, 2020, 46(11): 18281-18286.
9. Y. Li, L.L. Liu, J.X. Zuo, S.B. Liu, F.L. Yang, J.Q. Peng, Y.M. Yang, X.Y. Ye, Treatment with Na_2_SO_3_ alkaline reductant to restore luminescence intensity and improve the moisture resistance of deteriorated Mn^4+^-doped fluoride phosphors, J. Mater. Sci. 57 (2022) 15737–15751.
10. Qiang J, Wang L, Wang T, et al. Improvement of the luminescent thermal stability and water resistance of K_2_SiF_6_:Mn^4+^ by surface passivation[J]. Ceramics International, 2022, 48(12): 17253-17260.
11. Y.X. Liu, J.X. Hu, L.C. Ju, C. Cai, V.B. Hao, S.H. Zhang, Z.W. Zhang, X. Xu, X. Jian, L.J. Yin, Hydrophobic surface modification toward highly stable K_2_SiF_6_:Mn^4+^ phosphor for white light-emitting diodes, Ceram. Int. 46 (2020) 8811–8818.
12. L. Huang, Y. Liu, J. Yu, Y. Zhu, F. Pan, T. Xuan, M.G. Brik, C. Wang, J. Wang, Highly stable K_2_SiF_6_:Mn^4+^@K_2_SiF_6_:Mn^4+^ composite phosphor with narrow red emission for white LEDs, ACS Appl Mater Interfaces 10 (2018) 18082–18092.
13. P. Arunkumar, Y.H. Kim, H.J. Kim, S. Unithrattil, W.B. Im, Hydrophobic organic skin as a protective shield for moisture-sensitive phosphor-based optoelectronic devices, ACS Appl. Mater. Interfaces. 9 (2017) 7232–7240.
14. L.L. Liu, D. Wu, S.G. He, Z.J. Ouyang, J.F. Zhang, F. Du, J.Q. Peng, F.L. Yang, X. Y. Ye, A Reverse strategy to restore the moisture-deteriorated luminescence properties and improve the humidity resistance of Mn^4+^-doped fluoride phosphors, Chem. Asian J. 15 (2020) 3326–3337.
15. Luo P, Ye M, Zhou W, et al. Simultaneous construction of impermeable dual-shell stabilizing fluoride phosphors for white light-emitting diodes[J]. Chemical Engineering Journal, 2022, 435: 134951.
16. Pan X, Wu D, Liu S, et al. A novel thiourea loading strategy for improving the moisture resistance of K_2_SiF_6_:Mn^4+^ red phosphors[J]. Materials Research Bulletin, 2023, 163: 112215.
17. Wan P, Liang Z, Luo P, et al. Reconstruction of Mn^4+^-free shell achieving highly stable red-emitting fluoride phosphors for light-emitting diodes[J]. Chemical Engineering Journal, 2021, 426: 131350.
18. Ruan H, Wang T, Wang L, et al. Ion exchange-promoted surface reduction strategy: For improving the water-resistance of K_2_SiF_6_:Mn^4+^ red phosphors[J]. Ceramics International, 2023, 49(22): 35165-35174.
19. Chang C, Ye W, Zuo C, et al. Highly Moisture-Stable and Enhanced Luminescence-Efficient Mn^4+^-Activated Red-Emitting Fluoride Phosphors via a Bi-hydrogen-Bond Organic Coating[J]. ACS Sustainable Chemistry & Engineering, 2023, 11(44): 15887-15897.
